# Current Status of Comprehensive Chromosome Screening for Elective Single-Embryo Transfer

**DOI:** 10.1155/2014/581783

**Published:** 2014-06-01

**Authors:** Ming-Yih Wu, Kuang-Han Chao, Chin-Der Chen, Li-Jung Chang, Shee-Uan Chen, Yu-Shih Yang

**Affiliations:** Department of Obstetrics and Gynecology, National Taiwan University Hospital and College of Medicine, 7 Chung-Shan South Road, Taipei 10002, Taiwan

## Abstract

Most *in vitro* fertilization (IVF) experts and infertility patients agree that the most ideal assisted reproductive technology (ART) outcome is to have a healthy, full-term singleton born. To this end, the most reliable policy is the single-embryo transfer (SET). However, unsatisfactory results in IVF may result from plenty of factors, in which aneuploidy associated with advanced maternal age is a major hurdle. Throughout the past few years, we have got a big leap in advancement of the genetic screening of embryos on aneuploidy, translocation, or mutations. This facilitates a higher success rate in IVF accompanied by the policy of elective SET (eSET). As the cost is lowering while the scale of genome characterization continues to be up over the recent years, the contemporary technologies on trophectoderm biopsy and freezing-thaw, comprehensive chromosome screening (CCS) with eSET appear to be getting more and more popular for modern IVF centers. Furthermore, evidence has showen that, by these avant-garde techniques (trophectoderm biopsy, vitrification, and CCS), older infertile women with the help of eSET may have an opportunity to increase the success of their live birth rates approaching those reported in younger infertility patients.

## 1. Introduction

In a fresh IVF cycle, single-embryo transfer (SET) is associated with a lower rate of multiple pregnancies than other principles of embryo transfer. For this reason, SET became more popular in the past decade and had a good perinatal outcome in the US [1], in the Nordic countries [2], and even in Asia in recent years [3]. A number of factors are responsible for the variation in the practice of SET, such as advanced maternal age, legislation, and economic factors, all of which also play an important role in predicting favorable outcome of assisted reproductive technology (ART), including SET, to both physicians and patients. By and large, transfer of a good blastocyst by embryo grading of the Society for Assisted Reproductive Technology (SART) will produce a good implantation rate and live birth rate [4].

In addition to morphological evaluation, several “Omics” technologies, including genomics, transcriptomics, proteomics, and metabolomics, can also be employed to evaluate the implantation potential of an embryo. For instance, Seli et al. [5] used infrared spectroscopy to analyze the conditioned media from human embryos by metabolomic profiling and showed good correlation with the embryo implantation independent of morphology. Katz-Jaffe et al. have demonstrated abnormal elevation of embryonic secretome in aneuploidy embryos [6, 7]. Moreover, using transcriptome assay, direct measurement of granulocyte colony-stimulating factor (G-CSF) in the follicular fluid of individual oocytes was found to well correlate with the potential for an ongoing pregnancy [8]. Recently, a model of pregnancy prediction in SET was built on selected quantified transcripts in cumulus cells, which participate in the decision of embryo selection [9]. Several differentially expressed miRNAs between euploid and aneuploidy embryos were also confirmed by real-time quantitative PCR (qPCR) [10].

Since the first attempt to karyotype embryos 30 years ago [11], several techniques, such as fluorescence* in situ* hybridization (FISH), comparative genomic hybridization (CGH), array CGH (aCGH), digital PCR (dPCR), single-nucleotide polymorphism (SNP) array, qPCR, and next-generation sequencing (NGS), have been developed to analyze 24-chromosome copy number in human embryos clinically [12]. In this paper, we will review the accuracy and efficiency of those technologies for clinical use.

## 2. Single-Embryo Transfer

For younger women, SET is an option with a similar success rate to multiple embryo transfer. However, older women (>38 years old) had significantly lower pregnancy rates and seldom chose SET [1]. In a recent study on women aged 40–44 years in Finland, the researchers compared the outcomes in groups of elective single-embryo transfer (eSET) and double-embryo transfer (DET) [13]. They found that there were similar clinical pregnancy rates, live birth rates, and miscarriage between eSET and DET in fresh cycles, but eSET had higher clinical pregnancy rates and live birth rates than those of DET in cumulative results. The unfavorable outcomes in older women, such as miscarriage or IVF failure, were mainly a consequence of the increased number of aneuploidy [14]. A recent report revealed that aneuploidy of embryos increased predictably after 26 years of age. Notably by the age of 44, 88.2% of women's embryos were aneuploid; namely, more than 50% patients would barely have an euploid embryo to transfer [15]. In contrast to transcriptomics, proteomics, or metabolomics, embryo biopsy followed by genomic analysis could provide direct evidence of aneuploidy.

## 3. Fluorescence* In Situ* Hybridization

The first molecular cytogenetic technique to be applied in comprehensive chromosome screening (CCS) is FISH combined with chromosome-specific probes labeled with different fluorochromes. Although, via washing technique, multicolor FISH can detect 5–9 probes, the accuracy and efficiency would decline rapidly. Since FISH can analyze only a limited number of chromosomal loci, some of the embryos transferred might be diagnosed as “normal” but in fact be aneuploid for one or more chromosomes not tested. So recent advance will focus on analyzing all 24 chromosomes [16].

However, in 2007, a multicenter, randomized, double-blind, controlled trial comparing three cycles of IVF with and without preimplantation genetic screening (PGS, using FISH probe on chromosomes 1, 13, 16, 17, 18, 21, *X*, and *Y*) in women 35 to 41 years of age was conducted. It showed FISH did not increase but instead significantly decreased the rates of ongoing pregnancies and live births after IVF [17]. Several mechanisms were proposed. First, the biopsy on day 3 may hamper the embryo's potential. Second, limited chromosome number detected cannot promise normal embryo. Third, mosaic embryos from IVF exist substantially. Regarding the mosaicism, a Belgian series recently reported its incidence as high as 71.4% in human preimplantation embryos with good quality [18]. At present, there are numerous studies indicating that aneuploidy diagnosis in morphologically normal blastocysts is poorly predicted by cleavage stage FISH. The differences between the cleavage and blastocyst stages (including mosaicism, self-correction of aneuploidy) were explained by the preferential segregation or confinement of aneuploidy to the trophectoderm (TE) [19].

## 4. Comparative Genomic Hybridization

CGH, a technique that emerged in 1992 [20], has proved to be a powerful tool for molecular cytogenetic analysis of neoplasms. It provides an overview of DNA sequence copy number changes (losses, deletions, gains, and amplifications) in a tumor specimen and also maps these changes on normal chromosomes. At present, CGH is a research tool that complements previous methods for genetic analysis, for example, CCS before embryo transfer in an IVF cycle. For isolated single cells, whole genome amplification (WGA) is necessary to provide enough DNA for subsequent PCRs [21, 22].

It is performed by competitive fluorescence* in situ* hybridization. DNA isolated from the samples and reference was independently labeled with different fluorophores. DNA were then denatured to single stranded conformation, and a 1 : 1 ratio mix of two sources was hybridized to a normal metaphase spread of chromosome (CGH-metaphase spreads). Microscopic inspections were performed along the length of each chromosome for identification of chromosomal sections displaying difference of fluorescence intensities. It reflects its relative copy number in the test genome compared with the control genome. In 1999, Wells et al. have successfully used CGH to analyze single blastomere from human preimplantation embryos [23]. Using this CGH technique followed by a frozen embryo transfer (FET), a healthy infant was born to a woman with a history of implantation failure in 2001 [24]. However, this method is time consuming (up to 72 hours) and labor intensive. When a blastocyst biopsy is performed, it will need embryo freezing and will therefore delay transfer. In addition, the sensitivity is limited for traditional CGH (5–10 Mbp). Recently, a faster (12-hour protocol) and more sensitive method (detecting translocation) was developed to improve these shortcomings [25, 26].

## 5. Array CGH

A new technique, microarray-based CGH, has been developed to increase the diagnostic accuracy and efficiency [27, 28]. Now the aCGH provides 24-chromosome analysis to screen the translocation and all the other aneuploidies rather than a set of 5–12 chromosomal probes used by traditional FISH method. It allows automation in data reading through computerized calculation of signal intensities, not observing the signals by eye as in the FISH method. So, aCGH method is robust (2.9% no results) with high specificity (1.9% error rate) when applied to rapid (24-hour) analysis of single cells biopsied from cleavage-stage embryos [29]. The first birth after preimplantation diagnosis (PGD) of structural chromosome abnormalities using aCGH was reported in 2011 [30]. Recently, it has been demonstrated that aCGH for cleavage stage PGS is a feasible and safe option for aneuploidy screening that shows excellent outcomes when used in fresh cycles [31]. In fact, the influence of advanced maternal age (up to 42 years) on implantation of IVF was diminished after implementing aCGH screening [32].

Although CGH and aCGH performed well on many types of embryonic material tested (PBs, blastomeres, and trophectoderm), there is still debate over the efficacy of day-3 (blastomere) biopsy. Cell biopsy at the cleavage stage involves the loss of a significant proportion of the embryo volume, potentially impacting viability [33]. Now, we can routinely retrieve more genetic materials from blastocyst biopsy, providing more reliable results [34], and aCGH offers not only more comprehensive analysis of 24 chromosomes than traditional FISH but also shorter test duration (12–24 hours) to transfer in fresh cycles.

## 6. Digital PCR

The principle of dPCR is based on partitioning a sample into many individual real-time PCR reactions; some portions of these reactions contain the target molecule (positive), while others do not (negative). It is not dependent on the number of amplification cycles to determine the initial sample amount, but it provides absolute quantification of DNA template. Applications include copy number variation, aneuploidy detection, rare sequence detection, mutation detection, and gene expression analysis. So dPCR was frequently applied for illness such as cancer, in which molecular copy-number counting is vital [35]. Also, it has been used for detection of aneuploidy (e.g., trisomy 21) of fetal cells in maternal serum [36].

Recently, a more advanced technique, droplet digital PCR (ddPCR), was developed for very low copy event detection [37]. It is a digital PCR method utilizing a water-oil emulsion droplet for partitioning of DNA template. The droplet generator uses microfluidics to partition each sample into 20,000 water-in-oil nanoliter droplets. It offers a higher level of partitioning at a low cost compared to the traditional dPCR. ddPCR can be also used as a tool to precisely measure HER2 copy number alterations in formalin-fixed paraffin-embedded tissue of breast cancer [38].

## 7. SNP Array

Like aCGH, a SNP array contains a high number of probes in order to study the whole genome or to target specific regions. Up to date, almost 50 million SNPs have been identified in the human genome. In SNP arrays, a patient's DNA is not cohybridized with a DNA control but the fluorescent signal intensity of each probe is compared with a reference bioinformatic file. SNP arrays can be used to detect loss of heterozygosity (LOH) in which one allele of a gene is lost and results in loss of normal function, for example, tumor suppressor gene. In addition, SNP array is able to detect copy-neutral LOH in which one allele or whole chromosome from a parent is missing and disease may occur.

For aneuploidy screening, Treff et al. have validated SNP array with over 99% accuracy on single cells [39] and it is significantly more consistent than FISH on 24-chromosome aneuploidy screening [40]. Later, this group conducted a prospective, double-blinded, randomized study in which a total of 255 IVF-derived human embryos were cultured and selected for transfer independent of CCS analysis. They noted that CCS by SNP array was highly predictive for aneuploidy screening and well correlated with clinical outcome [41]. They also applied this technique in trophectoderm biopsy followed by FET and achieved a very high implantation rate (65%) and live birth rate (73%) [42].

For translocation detection, SNP array has been used to detect the chromosome imbalance and improve outcomes for these couples carrying translocations [43, 44]. Rabinowitz et al. also used SNP array-based genotyping and informatics-based techniques to characterize the origins and rates of aneuploidy in human blastomeres [45]. They showed the rate of maternal meiotic trisomy rose significantly with age, whereas other types of trisomy showed no correlation with age. In addition to monogenic disease, aneuploidy, and imbalanced translocation, commercial SNP arrays services such as 23andMe could allow for analysis of other multifactorial diseases, such as diabetes or heart disease.

## 8. Real-Time Quantitative PCR

In 2012, Treff et al. developed a quantitative real-time PCR- (qPCR-) based method for blastocyst trophectoderm and got 98.6% identical diagnosis with SNP array [46]. The overall euploidy and aneuploidy were assigned with 100% consistency. With this method, a preamplification step is used to amplify at least two sequences on each arm of each chromosome (1.5 hours). Then real-time qPCR is used for the rapid quantification of each product (2 hours). This qPCR was capable of accurate aneuploidy screening of all 24 chromosomes in 4 hours and could provide an opportunity to evaluate the trophectoderm biopsies with subsequent fresh euploid blastocyst transfer [47]. Due to this rapid, real-time qPCR technique, it alters clinical management: traditional morphology-based embryo selection [48].

There is no need for WGA in qPCR and the number of DNA probe is low. It is fast and of low cost compared to aCGH or SNP array. The only limitation of this technology is the limited number of samples, currently two on each plate, which can be run on the available equipment. However, excellent outcomes may be achieved by vitrifying all tested blastocysts and sending biopsy samples to a reference laboratory for qPCR examination.

## 9. Next-Generation Sequencing

The high demand for low-cost sequencing has driven the development of next-generation sequencing (NGS) technologies that run the sequencing process in parallel. It provides novel high-throughput, highly parallel, and base-pair resolution data for genetic analysis. Prior to NGS technology, genomics only investigated genomes which were feasible from the standard point of size and with characterization of single genes related to diseases. Between 2008 and 2010, the 1000 Genomes Project has built the 1,092 haplotype-resolved genomes [49]. In the era of NGS, sequencing now enables clinical diagnostics and other aspects of medical care, including disease risk, therapeutic identification, and prenatal testing.

In 2011, one remarkable study using NGS has shown the clinical and analytic validity of the targeted exome sequencing of a preselected group of genes that are known to be associated with severe pediatric onset autosomal recessive diseases including Tay-Sachs disease (OMIN#272800) and cystic fibrosis (OMIN#219700) [50]. For PGD, Yin et al. introduced a method of massively parallel sequencing for aneuploidy of blastocyst and showed 68.4% euploidy rate [51]. Those results were confirmed by SNP array and produced 97.4% consistency. More recently, Treff et al. used NGS to diagnose single gene disease (SGD) and validated it as a 100% equivalent diagnosis to qPCR [52]. Moreover, by increasing the read depth, NGS can provide accurate sequencing information of mutation site.

## 10. Conclusion

The universal goal of assisted reproduction technologies is a singleton delivery of a healthy full-term baby. Thus far, SET is the most effective strategy to prevent multiple pregnancies. However, advanced maternal age is a critical determinant for IVF efficiency to most ART centers. According to a recent review, the aneuploid percentage was over 50% if a woman was older than 38 years of age due to a failure of embryo implantation [15]. To provide an effective SET option for older women, improved methods of embryo selection by CCS are required [53].

Genomics is a relatively new scientific discipline, having DNA sequencing as its core technology. Today, with the far and quick advance of molecular genetic technologies, we are able to analyze the “24 chromosomes” and, moreover, the single gene polymorphism, translocation, or SGD [54]. In this study, we have reviewed the available methods of CCS for 24 chromosomes and compared their power and limitations (Table 1). However, final assessment, which includes live birth rates per cycle commenced, will be most important in the clinical evaluation of CCS.

## Figures and Tables

**Table 1:** Comparison of available CCS for 24 chromosomes.

	aCGH	dPCR	SNP array	Real-time qPCR	NGS
Test time (hours)	12–24	8	16–72	4	15
Single gene disease	Y^#^	Y*	Y*	Y*	Y
Resolution	Medium	Low	High	Low	High
WGA needed	Y	N	Y	N	Y
Probe number	2~32 K	~20	262~370 K	96	3 × 10^9^
Posttest algorithm	Moderate	Easy	Moderate	Easy	Intensive
Cost ($)	High	Medium	High	Low	Very high

^#^Only deletion/duplication can be detected; *with known mutations.
